# Potential therapeutic targets shared between leishmaniasis and cancer

**DOI:** 10.1017/S0031182021000160

**Published:** 2021-05

**Authors:** Sajad Rashidi, Celia Fernández-Rubio, Raúl Manzano-Román, Reza Mansouri, Reza Shafiei, Mohammad Ali-Hassanzadeh, Afshin Barazesh, Mohammadreza Karimazar, Gholamreza Hatam, Paul Nguewa

**Affiliations:** 1 Department of Parasitology and Mycology, School of Medicine, Shiraz University of Medical Sciences, Shiraz, Iran; 2 Department of Microbiology and Parasitology, IdiSNA (Navarra Institute for Health Research), c/ Irunlarrea 1, University of Navarra, ISTUN Instituto de Salud Tropical, 31008 Pamplona, Spain; 3 Proteomics Unit, Cancer Research Centre (IBMCC/CSIC/USAL/IBSAL), 37007 Salamanca, Spain; 4 Department of Immunology, Faculty of Medicine, Shahid Sadoughi University of Medical Sciences and Health Services, Yazd, Iran; 5 Vector-borne Diseases Research Center, North Khorasan University of Medical Sciences, Bojnurd, Iran; 6 Department of Immunology, School of Medicine, Jiroft University of Medical Sciences, Jiroft, Iran; 7 Department of Microbiology and Parasitology, Faculty of Medicine, Bushehr University of Medical Sciences, Bushehr, Iran; 8 Basic Sciences in Infectious Diseases Research Center, Shiraz University of Medical Sciences, Shiraz, Iran

**Keywords:** Cancer, common protein, drug, *Leishmaniasis*, therapeutic target

## Abstract

The association of leishmaniasis and malignancies in human and animal models has been highlighted in recent years. The misdiagnosis of coexistence of leishmaniasis and cancer and the use of common drugs in the treatment of such diseases prompt us to further survey the molecular biology of *Leishmania* parasites and cancer cells. The information regarding common expressed proteins, as possible therapeutic targets, in *Leishmania* parasites and cancer cells is scarce. Therefore, the current study reviews proteins, and investigates the regulation and functions of several key proteins in *Leishmania* parasites and cancer cells. The up- and down-regulations of such proteins were mostly related to survival, development, pathogenicity, metabolic pathways and vital signalling in *Leishmania* parasites and cancer cells. The presence of common expressed proteins in *Leishmania* parasites and cancer cells reveals valuable information regarding the possible shared mechanisms of pathogenicity and opportunities for therapeutic targeting in leishmaniasis and cancers in the future.

## Graphical abstract

**Figure:**
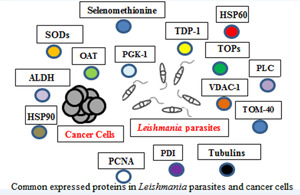

## Introduction

### Leishmaniasis and cancer

Leishmaniasis is a group of vector-borne diseases caused by intracellular protozoan belonging to the genus *Leishmania*. Annually, approximately 1.5–2 million new cases are reported worldwide being 310 million people at risk. The mortality rate of the disease varies from 40 000 to 70 000 cases per year (Torres-Guerrero *et al*., 2017). Clinical manifestations vary depending on the *Leishmania* species and the immune status of the host, among others. The clinical symptoms of cutaneous leishmaniasis (CL) are mostly restricted to the skin lesions with diverse appearances, from localized to body extended wounds or mucosal affectations. However, visceral leishmaniasis (VL) is characterized by severe organic symptoms and might lead to death (Den Boer *et al*., 2011; Torres-Guerrero *et al*., 2017). Malnutrition, acquired immune deficiency syndrome and cancer are important factors that affect the host immune system and lead to more severe clinical symptoms in patients with leishmaniasis (Ezra *et al*., 2010; Nweze *et al*., 2020).

Cancer is a group of diseases involving an abnormal growth of cells, which tend to proliferate in an uncontrolled way and, in some cases, metastasize. It can affect almost any tissue of the body. After coronary artery diseases, cancer is the second major cause of death in humans. Each year, the global mortality rate of cancers is estimated at 8.2 million deaths and approximately 14.1 million new cases are being reported (Torre *et al*., 2016). The clinical manifestations of cancers are wide-ranging and the immunosuppression is a critical side-effect to be considered during the management of cancers (Blagosklonny, 2013).

## The possible association of leishmaniasis and malignancies (cancers)

Although smoking is one of the principal causes of cancer development, infections are also a risk factor, mainly those caused by bacteria (*Helicobacter pylori*) (Nguewa *et al*., 2016) and viruses (*Human papillomavirus*, *Hepatitis B* and *C viruses*, *Herpes virus*, *Epstein–Bar virus* and *human T-cell leukaemia-lymphoma virus*) (Liao, 2006). However, certain parasitic infections (by *Opisthorchis*, *Clonorchis*, *Theileria* and *Schistosoma*) can also raise the risk of developing some types of cancers and may contribute to the appearance of malignancies which makes them possible models to study host–parasite interactions and mechanisms of cancer (De Martel *et al*., 2012; Tretina *et al*., 2015; Cheeseman and Weitzman, 2017).

The association of leishmaniasis and malignancies in human and animal models has been highlighted in previous studies (Kopterides *et al*., 2007; Ferro *et al*., 2013; Al-Kamel, 2017). Due to the relatively similar clinical manifestations in certain leishmaniasis forms and cancers, misdiagnosis might occur in the clinic (Toogeh *et al*., 2010; Schwing *et al*., 2019). For instance, the diagnosis of childhood leukaemia should be carefully differentiated from VL, especially in endemic areas where the concurrent occurrence had been reported (Vasconcelos *et al*., 2014). Similarly, cutaneous and mucocutaneous leishmaniasis may be clinically misdiagnosed as squamous cell carcinoma (SCC) (Ramos *et al*., 2015; Oetken *et al*., 2017). These data point out the possible similar association in clinical manifestations of leishmaniasis and tumoural alterations. Moreover, failures at epigenetic level to maintain integrity of chromosomes is one contributing factor in cancer and *Leishmania* parasites also modulate and destabilize the host chromatin structure leading to potential changes in relevant immune-related genes and responses (Sarkar *et al*., 2015; Afrin *et al*., 2019; Dacher *et al*., 2019).

In addition, numerous compounds with anti-tumour activity have exhibited potent leishmanicidal properties (Table 1). The use of common drugs for the treatment of leishmaniasis and cancer might further propose and highlight the presence of plausible similarities in their molecular mechanisms of action, immunopathobiology and therapeutic targets in both diseases (Table 2) (Kopterides *et al*., 2007; Miguel *et al*., 2007, 2008; Moulisha *et al*., 2010; Toogeh *et al*., 2010). Furthermore, chemotherapies administered against some forms of cancer display immune dysfunctions and/or immunosuppression. An example is bortezomib, a proteasome inhibitor which decreases the dendritic cells’ (DCs) activity, the number of T lymphocytes and interferon (IFN) gamma production (Nucci and Anaissie, 2009). This immunosuppressive effect may lead to leishmaniasis development as an opportunistic infection in antitumour-treated patients but also in immunocompetent ones leading to similar symptoms (Piro *et al*., 2012; Cencini *et al*., 2015; Torti *et al*., 2015; Schwing *et al*., 2019). Additionally, a synergistic relationship between *Leishmania* parasites and cancer cells has been highlighted (Morsy, 2013). Now, case reports and clinical observations suggest that leishmaniasis may be a risk factor for certain cancers and that cancer immunosuppression may facilitate *Leishmania* infections (Morsy, 2013; Liao *et al*., 2018; Nicolas *et al*., 2018; Carrillo-Larco *et al*., 2019; Claudio *et al*., 2019). However, a comprehensive review regarding common expressed proteins in *Leishmania* parasites and cancer cells is lacking. Therefore, reviewing and highlighting such functional proteins might reveal valuable information regarding the possible shared mechanisms of pathogenicity and possible therapeutic targets in leishmaniasis and cancers in the future.

**Table 1.** Some common compounds used against cancer and leishmaniasis

Anti-leishmaniasis and -cancer drugs	Possible drug action in leishmaniasis (or protozoan parasites)	Possible drug action in cancer	References
Dichloroacetic acid (DCA)	Switching infected-cell metabolism from aerobic glycolysis to oxidative phosphorylation by restoring mitochondrial activity	Inhibition of pyruvate dehydrogenase kinase (PDK) (leading to pyruvate dehydrogenase (PDH) activation and fostering oxidative phosphorylation)/the increase of each Krebs cycle intermediate concentration/inducing cell toxicity through *de novo* synthesis of CoA/modulating intracellular acidification/inhibition of Na-K-2Cl cotransporter	Tataranni and Piccoli (2019); Martínez-Flórez *et al*. (2020)
3-Bromopyruvic acid (3BP)	Blocking early stages of the glycolytic cycle (inhibition of HK II or GAPDH activity)	Inducing cell death by necroptosis and apoptosis	Sun *et al*. (2015); Martínez-Flórez *et al*. (2020)
Diospyrin	Inhibitor of type I DNA TOP of the parasite Promoting apoptotic death of the infected cell	Targeting several genes and pathways in the cell (affecting cell proliferation and survival)/targeting cell transcriptional level/possible targeting of TOPs	Ray *et al*. (1998); Sagar *et al*. (2010)
Modified lapachones	Inhibition of serine proteinase activity in parasite	Nicotinamide adenine dinucleotide (NAD^+^) depletion	Souza-Silva *et al*. (2015); Silvers *et al*. (2017)
4-Substituted quinoline derivatives	Inhibitory activity against cysteine proteases type B	The occurrence of caspase-dependent apoptosis with involvement of mitochondrial permeabilization/inhibition of cysteine proteases activity	Costa *et al*. (2020)
DL-*α*-difluoromethylornithine (DFMO)	Inhibitor of ornithine decarboxylase		Schechter *et al*. (1987); Keithly and Fairlamb (1989)
17-*N*-Allylamino-17-demethoxygeldanamycin (17-AAG, tanespimycin)	Inhibitor of heat shock protein 90 (HSP90)		Petersen *et al*. (2018); Pires *et al*. (2020)
Gold(I) complexes with 1,3,4-oxadiazole-2(3*H*)-thione ligands derived from *δ*-D gluconolactone	Acting as anti-inflammatory agents and help immune response to tackle both tumour cells and *Leishmania* parasites		Espinosa *et al*. (2020)

**Table 2.** Possible functions of several common proteins expressed in *Leishmania* parasites and cancer cells and plausible inhibitors/drugs against such proteins

Cells	Proteins and possible functions										
*Leishmania*	HSPs		PDI	PL	ALDH	VDAC-1	PCNA	Tubulins	SODs (Fe-SOD)	TOPs	
	HSP60	HSP90								TOP IB	TOP II
	Pathogenicity	Survival and proliferation	Pathogenicity and survival	PLC: pathogenicity PLA2: virulence	Virulence and protective function against oxidative stress	Survival and adaptation to the environmental stresses	Pathology of the parasite (significant role in drug response)	Involved in parasite shape, replication, motility and pathology (drug resistance strains)	Protection against radical superoxide anions	Cut one DNA strand and covalently join to 3′ end	Catalyse topological changes in the DNA
Inhibitor	Suramin	Geldanamycin, 17-DMAG, 17-AAG, Glb02, Glb08, Glb11, Glb16, Glb25, Glb27	Bacitracin	PLA2: BEL	Gossypol	Ion channel blockers: verapami, nifedipine, and ZINC29590262	Selenocompounds	Selenocompounds, podophyllum-derived toxins	2-Methoxyoestradiol and synthesized selenium derivatives	Camptothecins, indenoisoquinolines, natural alkaloids and marine fatty acids	Isobenzofuranone derivatives, protoberberine, mitonafide
Cancer	Survival in neuroblastoma cells, anti-tumour in in human hepatoma Huh-7 cells	Tumour initiation, development and metastasis	Survival, progression and metastasis	PI-PLC-*β*1: progression of MDS PLC*γ*1: development of OPL	Pathogenicity and drug resistance	Survival and apoptosis	DNA replication and repair that is often overexpressed in cancer cells	Cell cycle promotion and drug resistance in cancers	Anti-tumour in brain malignant tumours Progression and metastasis of pancreatic cancer	TOP IB: cut one DNA strand and covalently join to 3′ end	TOP II: catalyse topological changes in the DNA
Inhibitor	Epolactaene, bortezomib, sinularin and myrtucommulone	Geldanamycin, 17-DMAG, 17-AAG	PACMA, 16F16, bacitracin and RB-11-ca	Phosphatidylcholine-specific PLC inhibitor: D609	DEAB, Gossypol in combination with phenformin	Ion channel blockers: verapami and nifedipine	A cell-penetrating AlkB homologue 2 PCNA-interacting motif (APIM)-containing peptide: ATX-101	Microtubule-destabilizing tubulin inhibitors: colchicine analogues and vinca alkaloids Microtubule-stabilizing tubulin inhibitors: taxanes (paclitaxel derivatives)	DDC, ATN-224 and 2-methoxyoestradiol	Camptothecins (irinotecan, topotecan and belotecan)	Etoposide: anthracycline and podophyllotoxin derivatives

## Drugs for the treatment of leishmaniasis and cancer with common signalling processes

Drug repurposing is an extensively strategy used to identify new microbicidal compounds including leishmanicidal agents. The effect of antitumour chemical compounds in leishmaniasis treatment might suggest the existence of possible similar mechanisms of pathogenicity and common therapeutic targets in *Leishmania* parasites and cancer cells. Selenocompounds have demonstrated antitumour properties blocking the mammalian target of rapamycin (mTOR) pathway (Ibanez *et al*., 2012) and reduced *Leishmania* parasite burden during ‘*in vitro*’ assays. Furthermore, these compounds were able to reduce the expression of leishmanial genes involved in cell cycle, resistance to treatment and virulence at the mRNA level (Fernández-Rubio *et al*., 2015). Similarly, naphtilamide derivatives which were previously synthesized as antitumour agents (Karelia *et al*., 2017), decreased intracellular amastigotes burden, caused cell cycle arrest and diminished the topoisomerase-2 (TOP II), mini-chromosome maintenance complex (MCM4) and proliferating cell nuclear antigen (PCNA) mRNA levels (Fernández-Rubio *et al*., 2019). Anti-cancer and anti-leishmaniasis effects of herbal compounds such as pentacyclic triterpenoid are well known (Moulisha *et al*., 2010). Fatty acids from natural sources are inhibitors of therapeutic targets in cancer cells and *Leishmania* parasites (Carballeira *et al*., 2011, 2018; Carballeira, 2013).

Moreover, a set of compounds described as antitumoural drugs also exhibited leishmanicidal activities. Alkylating antineoplastic agents such as cisplatin are inductors of cell death in both, tumour cells and parasites (Fuertes *et al*., 2003; Nguewa *et al*., 2005). Miltefosine (hexadecylphosphocholine, HePC), an alkyl phospholipids compound, has been originally considered for breast cancer and other solid tumours’ treatment. Two compounds of the alkylphosphocholine group, octadecyl-phosphocholine and hexadecylphosphocholine-miltefosine (HePC), have been found to have antineoplastic activity. The mechanism of antitumour action of these compounds was involved in the inhibition of substrate phosphorylation by protein kinase C (PKC) [triggering programmed cell death (apoptosis)]. The presence of PKC on *Leishmania* membrane led to the further investigations on such compounds against CL. It has been indicated that miltefosine inhibits the biosynthesis of the glycosyl phosphatidyl inositol receptor, a vital molecule for *Leishmania* intracellular survival. Moreover, this compound interferes with the synthesis of leishmanial-phospholipase (PL) and PKC. The metabolic action of miltefosine affects the biosynthesis of glycolipids and membrane glycoproteins of the *Leishmania* parasite, leading to apoptosis (Sundar and Olliaro, 2007; Perez *et al*., 2008; dos Santos Nogueira *et al*., 2019; Fernández-Rubio *et al*., 2019). Another example is tamoxifen (as a triphenylethylene), a breast cancer drug that has shown an appropriate efficacy in the treatment of leishmaniasis (Miguel *et al*., 2007, 2008). Due to the activity of tamoxifen as an oestrogen receptor modulator, this drug has been used in the prevention and treatment of breast cancer. However, it has been elucidated that many biological effects of tamoxifen are independent of the oestrogen machinery, including modulation of calmodulin, kinases and caspases, inhibition of the acidification of intracellular organelles, interference in ceramide metabolism, and partitioning into lipids where it exerts membrane fluidizing and antioxidant activities. Oestrogen receptor-modulated responses are not present in *Leishmania* parasites. On the other hand, tamoxifen is able to inhibit the acidification of organelles in different cell types in an oestrogen-independent pathway. It seems that tamoxifen modifies the intravacuolar pH of *Leishmania*-infected macrophages inducing a condition where the drug activity against the *Leishmania* is increased (Miguel *et al*., 2007, 2008). In addition, camptothecin and its analogues target topoisomerase IB (TOP IB) and inhibit in *Leishmania* the activity of this relaxing enzyme for supercoiled DNA. Similarly, indenoisoquinolines, which are also TOP IB poisons, initially developed as antitumour compounds (Antony *et al*., 2005), are able to decrease parasite burden ‘*in vitro*’ and ‘*in vivo*’ in a mouse model (Balaña-Fouce *et al*., 2012). Furthermore, quinone derivatives that act as antitumour agents, showed anti-leishmanial activity through different mechanisms of action, including TOP II and trypanothione reductase inhibition (Sett *et al*., 1992; Mukherjee *et al*., 2004; Singh and Dey, 2007; Shukla *et al*., 2011).

## Immunological coincidences between leishmaniasis and cancer

Investigations have indicated the effect of *Leishmania* parasites on the immune system of patients with cancer thus triggering the modulation of anti-cancer immunity. In 2011, Kumar *et al*. highlighted the role of *Leishmania* in mutual modulation of the immune system in a patient with Hodgkin's lymphoma (Kumar *et al*., 2011). It's clear that in both diseases the host immune response is critical for the disease outcome. In this sense, immune checkpoints are essential for the regulation of the immune system homoeostasis and metastasis in cancer (Safarzadeh *et al*., 2020). These are also important factors regulating the function of T-cells and can be differentially modulated during pathogen infections (Cai *et al*., 2020). Leishmaniasis shares several key immunoregulatory features with cancer. Thus, in some forms of leishmaniasis, a number of important immune checkpoint molecules have also been identified (Kumar *et al*., 2017). Cytotoxic T-lymphocyte-associated protein 4 is one of the differentially induced immune checkpoints upon infection that may be targeted to ameliorate disease progression (Viana *et al*., 2019). These authors state that some *Leishmania* species induce a more inflammatory profile which also is well-established in cancer progression. Pattern recognition receptor expression and activation have pro-inflammatory effects on the tumour microenvironment *via* Toll-like receptor (TLR) signalling (McCall *et al*., 2020). *Leishmania* infections have differential capacities to activate TLRs and are able to block the TLR-based pro-inflammatory downstream signals. Parasite-derived microvesicles can activate specific TLR-based downstream immune inhibitory signals *via* CD200 to evade macrophage defenses and favour infection (Saha *et al*., 2019; Sauter *et al*., 2019).

Another shared mechanism is type I IFN-driven immunity. IFNs play essential roles in context-specific anti-pathogen responses and act in many immune-related processes in cancer including therapy (Silva-Barrios and Stager, 2017; Sprooten *et al*., 2019). Recently, it has been discovered that type I IFNs are involved in the negative regulation of CD4+ T cell responses in patients with VL in order to thus promote their persistence by suppressing anti-parasitic immunity (Kumar *et al*., 2020). It seems that the action of IFNs may be by targeting DCs to suppress consequently priming and/or expansion of Th1 specific cells during the infection. These authors have also stated with animal models the potential of targeting type I IFN signalling to strength protective immunity. In this regard, some molecules of *Leishmania major* are able to affect DC maturation thus potentially enhancing DC-based vaccines for cancer (Arab *et al*., 2019). Furthermore, live attenuated nonpathogenic *Leishmania* parasites directly may induce immune-stimulant responses to regress breast cancer grown (Caner *et al*., 2020). Interestingly, these attenuated parasites led to a higher percentage of CD4+ and CD8+ T-cells secreting pro-inflammatory cytokines tumour necrosis factor-*α* (TNF-*α*), interleukin-12 (IL-12), IFN-*γ*, inducible nitric oxide synthase and IL-2, and finally, inducing tumour cell death. Cancer therapy is an essential research area and protein kinases are major targets for drugs. The mTOR is a highly conserved serine/threonine protein kinase that is central regulating essential cellular processes and mTOR inhibitors are used in cancer therapy (Chen and Zhou, 2020). *Leishmania* infection activates this key host protein kinase pathway *via* its phosphorylation which have important effect towards host cellular physiology (M2 macrophage phenotype polarization) and parasite survival inside macrophages through down-regulation of the nuclear factor-*κ*B and signal transducer and activator of transcription 3 oncogene factors (Kumar *et al*., 2018). Another type of immune-related molecules which regulate diverse cellular processes involved in cancer and leishmaniasis are Ras, small cellular GTPases. Ras mutations are frequently observed in cancers and leukaemia leading to different isoforms. Currently, research data demonstrate that these isoforms are differentially activated by CD40 to modulate effector signals like inflammation playing crucial roles in infectious diseases and tumour regression between others (Nair *et al*., 2020). *Leishmania* infections are able to inhibit CD40-induced N-Ras activation as a survival strategy switching CD40 signalling leading to inhibition of the p38MAPK pathway, the master regulator of transcript stability and tumour progression (Chakraborty *et al*., 2015; Soni *et al*., 2019).

## From potential targets to drug repurposing

Novel shared targets may be identified by analysing proteins that play key roles in each disease. To date, mass spectrometry and functional proteomics along with other integrative-omics and multi-platforms allow shedding some light on the role of these molecules in different diseases (Cowell and Winzeler, 2019; Khan *et al*., 2020; Syu *et al*., 2020). In addition, some biomarkers of diseases seem to have high potential as drug targets, opening avenue for therapeutic screenings (Sharma *et al*., 2019; Roy *et al*., 2020). Accordingly, we therefore present some functional proteins that may be useful as shared/interchangeable therapeutic targets for the development pipeline of repurposed drugs against leishmaniasis and cancer.

### Protein disulphide isomerases (PDIs)

PDIs belong to a group of multifunctional proteins which are located in the endoplasmic reticulum and catalyse the formation of disulphide bonds during protein creation (Ben Khalaf *et al*., 2012). In *Leishmania* parasites, it has been reported the role of these proteins in pathogenicity and survival. Moreover, their potential immunostimulatory property suggests such proteins as possible drug and vaccine targets against leishmaniasis (Achour *et al*., 2002; Gupta *et al*., 2007; Ben Khalaf *et al*., 2012; Jaiswal *et al*., 2014; Amit *et al*., 2017).

On the other hand, the expression of PDIs can affect the survival, progression and metastasis of cancer. In fact, *PDI* gene is up-regulated in different cancer types such as lymphoma, brain, ovarian or kidney among others (Xu *et al*., 2014). PDI inhibitor developments from natural and synthetic compounds have demonstrated their effectiveness and cytotoxicity against tumours and *Leishmania* parasites (Ben Khalaf *et al*., 2012; Xu *et al*., 2014; Lee, 2017). For instance, propynoic acid carbamoyl methyl amides (PACMA) 31, a new irreversible PDI inhibitor, and 16F16, showed significant anticancer activity in *in vitro* and *in vivo* ovarian cancer models (Xu *et al*., 2012, 2014). The terminal propynoic group of PACMA covalently reacts with the thiol groups of the active-site cysteines in PDI. This interaction also changes the secondary protein structure of PDI. RB-11-ca, arsenic-containing compounds, sulphhydryl reagents, juniferdin and its analogues, quercetin-3-rutinoside, and bacitracin have been identified as possible PDI inhibitors against cancer cells (Xu *et al*., 2014). Among PDI inhibitor identified against cancer cells, bacitracin inhibited *in vitro* promastigote growth as well as amastigote propagation inside macrophages with EC_50_ values of 39 *μ*m. This compound blocked both reductase and isomerase activities of PDI in *Leishmania* parasite (Ben Khalaf *et al*., 2012).

### Superoxide dismutases (SODs)

SODs are considered potential cellular antioxidants in different cells since they are metalloproteins involved in the breakdown of potentially harmful oxygen molecules, preventing tissue damage. For instance, Fe-SOD protects *Leishmania* against radical superoxide anions using iron as a cofactor (Opperdoes and Szikora, 2006; Van Assche *et al*., 2011). SODs are proteins encoded by conserved genes in *Leishmania* parasites and due to their protective function, are considered possible therapeutic and vaccine targets against leishmaniasis (Paramchuk *et al*., 1997; Danesh-Bahreini *et al*., 2011; Sanchez-Moreno *et al*., 2015; Martin-Montes *et al*., 2017; Rashidi *et al*., 2020*a*).

Alternatively, the deregulation of the redox homoeostasis is implicated in several diseases, among them malignancies. It had been shown that the activity of Zn-SOD, Mn-SOD and Cu-SOD is decreased in cancer cells. Furthermore, in most brain malignant tumours, apoptosis occurs due to the expression of SODs (Younus, 2018) and are proteins inducing the progression and metastasis of pancreatic cancer cells (Oberley and Buettner, 1979). Due to the multifunctional role of these proteins, several inhibitors have been developed (Wood *et al*., 2001; Dumay *et al*., 2006; Glasauer *et al*., 2014). For instance, diethyldithiocarbamate (DDC) has been known as an inhibitor of Cu- and Zn-SODs. DDC has antagonistic effects on apoptosis by triggering cytochrome *c* release and caspase inhibition (Dumay *et al*., 2006). In addition, it has been indicated that SOD1 by the small molecule ATN-224 induced cell death in various non-small-cell lung cancers cells (NSCLCs). ATN-224-dependent SOD1 inhibition enhanced superoxide, which decreased the enzyme activity of the antioxidant glutathione peroxidase, causing an increase in intracellular hydrogen peroxide (H_2_O_2_) levels (Glasauer *et al*., 2014). 2-Methoxyoestradiol, a naturally occurring metabolic product of 17-beta-oestradiol, is able to inhibit tubulin polymerization and possesses growth inhibitory and cytotoxic activity *in vitro* and *in vivo*. 2-Methoxyoestradiol also inhibited SOD in a tetrazolium salt-based enzyme assay, proposed that oestrogen derivatives could be useful starting points for the development of non-toxic, effective enzyme inhibitors (Wood *et al*., 2001). In addition to application in cancer therapy, due to the expression of SODs in *Leishmania* parasites, the SOD inhibitory property of this compound can be evaluated against these parasites. In 2017, a series of synthesized selenium derivatives showed *in vitro* leishmanicidal activities against intracellular amastigote and promastigotes forms of *L*eishmania *braziliensis* and *Leishmania infantum* with significant low toxicity on the parasite-infected-macrophages. Surprisingly, the most active selenium compounds were potent inhibitors of Fe-SOD in both parasite species (Martín-Montes *et al*., 2017). Moreover, since tubulins are expressed in *Leishmania* parasites and considered appropriate drug targets, the tubulin polymerization inhibitory property of 2-methoxyoestradiol, is another factor that might underline this compound as a possible drug against leishmaniasis (Morgan *et al*., 2008).

### Phospholipase

PLC facilitates the evasion of protozoan parasites from parasitophorous vacuoles and helps to hydrolyse miltefosine in *Leishmania* parasites. Such critical functions highlight the role of PLC in the pathogenicity of *Leishmania* parasites (Breiser *et al*., 1987; Moudy *et al*., 2001; Dorlo *et al*., 2012; Rashidi *et al*., 2020*a*). Other PLs such as PLA2 play major roles in *Leishmania* parasites virulence and maintenance in vertebrate hosts. It has been indicated that the use of PLA2 inhibitor such as bromoenol lactone (BEL) led to the reduction of lesions size and decreased the load of parasites in skin in the *L. amazonensis*-infected BALB/c mice. However, the use of such an inhibitor also induced hepatotoxicity in BALB/c mice (Bordon *et al*., 2018).

PLCs as intermediate signalling factors for epidermal growth factor and ILs conduct regulatory functions in the immunology of cancers. However, the role of these proteins in the evasion of cancer cells from the host immune system remains unknown (Ramazzotti *et al*., 2011). The PLC-isoenzyme profile has not been investigated in *Leishmania* parasites so far. Nevertheless, the different forms of PLC-isoenzymes including PLC-*α*, PLC-*β*1, PLC-*ε* and PLC-*γ*1 have been characterized in breast cancer (Cai *et al*., 2017). It has been shown that nuclear phosphoinositide (PI)-PLC-*β*1 has a role in the generation, progression and resistance to apoptosis of the cancer cells in patients with myelodysplastic syndromes (MDS) (Ramazzotti *et al*., 2011). Other results highlighting the up-regulation of PLC-*γ*1 in the oral potentially malignant lesion (OPL) were correlated with the development of oral cancer (Ma *et al*., 2013). It was demonstrated that PLC-*γ* is an important marker in the pathogenicity of cancers (Lattanzio *et al*., 2013). The identification of PLC-isoenzymes in *Leishmania* parasites and the elucidation of possible functions of each PLC-isoenzyme regarding the pathogenicity and clinical manifestations of leishmaniasis might become a new approach for the development of leishmaniasis treatment. It has been shown that inhibition of phosphatidylcholine-specific PLC using tricyclodecan-9-yl-potassium xanthate (D609) selectively targeted proliferation and survival of tumour initiating cells in SCC and ovarian cancer cells. This compound prevented cancer cells from entering the S-phase under growth-factor stimulation without cell death induction (Amtmann and Sauer, 1990; Spadaro *et al*., 2008; Iorio *et al*., 2010; Cecchetti *et al*., 2015). Other compounds including aurintricarboxylic acid, 3013, 3017 and U73122 have been also identified as other possible PLC modulators (Bleasdale *et al*., 1990; Huang *et al*., 2013). These compounds can also be evaluated as inhibitors against PLCs in *Leishmania* parasites and cancer cells.

### Tyrosyl-DNA-phosphodiesterase-1 (TDP-1)

TDP-1 is a PL D able to cleave the phosphodiester bond formed between the tyrosine residue of type I TOP and the 3′ phosphate of DNA. TDP-1 is involved in repairing TOP I–DNA complexes stabilized by TOP IB poisons and performs its activity by hydrolysis of the phosphodiester bond (Banerjee *et al*., 2010). TDP-1 has been firstly described in *Leishmania donovani*. Recently, indenoisoquinoline derivative with dual TOP IB/TDP-1 inhibitory capability has been tested against *L. infantum* (Gutiérrez-Corbo *et al*., 2019).

It has been reported the altered expression of TDP-1 in several cancers (Liu *et al*., 2007; Dean *et al*., 2014; Meisenberg *et al*., 2015). Moreover, single nucleotide polymorphisms are associated with poor survival among small cell lung cancer patients (Lohavanichbutr *et al*., 2017). Due to its repair action mechanism, TDP-1 is related to resistance to TOP I inhibitors during cancer treatments. Therefore, its status in tumours might predict the effectiveness of the TOP I inhibitors used against cancers. TDP-1 constitutes a promising target in cancer treatment; therefore, the development of inhibitors may be useful to improve the efficacy of chemotherapy (Dean *et al*., 2014; Mozhaitsev *et al*., 2019; Khomenko *et al*., 2020; Mamontova *et al*., 2020).

### HSP60

HSPs categorize as a group of proteins that are regulated by different cells in response to exposure to stressful conditions. Several members of these proteins exert chaperone functions by facilitating to refold proteins that were destructed or damaged by the cell stress or by stabilizing new proteins to provide correct folding (Dubey *et al*., 2015). The immunostimulatory property of HSP60 and its up-regulation in *Leishmania*-infected cells and drug-resistant *Leishmania* strains lead to consider HSP60 as a valuable biomarker in the vaccination design and the treatment of leishmaniasis (Brandau *et al*., 1995; Celeste *et al*., 2004; Requena *et al*., 2015; Rashidi *et al*., 2019). During cancer development, HSP60 is overexpressed in advanced breast and serous ovarian cancers (Desmetz *et al*., 2008; Hjerpe *et al*., 2013). The expression of this protective protein leads to the angiogenesis, metastasis and survival of the cancer cells. Mostly, HSP60 exerts its functions by attaching other proteins. For instance, HSP60 promotes neuroblastoma cells survival through clusterin protein inhibition by a linkage to this protein. In addition, HSP60 binds to the *β*-catenin and induces metastasis in some cancer cells (Tsai *et al*., 2009; Chaiwatanasirikul and Sala, 2011). The regulation of apoptosis due to the interaction of HSP60 and cyclophilin D in mitochondrion suggests the dual function of HSP60 in cancer cells (Ghosh *et al*., 2010). In human hepatoma Huh-7 cells, through the interaction of HSP60 with the hepatitis C virus, core proteins induce the production of reactive oxygen species (ROS) and increase the apoptosis which is mediated by TNF-*α* (Sherman and Multhoff, 2007; Kang *et al*., 2009). Based on the importance of this chaperone in the viability of both *Leishmania* and cancer cells, and on the existence of inhibitors targeting them (Cappello *et al*., 2014; Stevens *et al*., 2019), HSP60 might be consider a promising therapeutic target against these pathologies. Three known antibiotics (suramin, rafoxanide and closantel) and epolactaene and myrtucommulone have been identified as inhibitors of human HSP60 chaperonin (Meng *et al*., 2018; Stevens *et al*., 2019). The use of suramin, as first-line chemotherapeutic agent, against *Trypanosoma brucei rhodesiense* and *T. brucei gambiense*, might suggest the evaluation of this HSP60 inhibitor against leishmanial-HSP60 (Abdeen *et al*., 2016; Zininga and Shonhai, 2019). Although the exact mechanism action of this compound remains unknown, probably inhibits some glycolytic enzymes (Willson *et al*., 1993; Zininga and Shonhai, 2019). Sinularin, a compound extracted from the coral Sinularia flexibilis, is able to inhibit HSP60 in melanoma cell-A2058 (Su *et al*., 2012). It has been also shown that bortezomib, a proteasome inhibitor, exhibited its anti-tumour efficacy by increasing HSP60 and HSP90 expression on the surface of cancer cells and inducing phagocytosis in experimental model of ovarian cancer (Chang *et al*., 2012).

### HSP90

HSP90 is a molecular chaperone important to the stability, folding and activity of over 200 proteins responsible for tumour initiation, development and metastasis. This protein is important for survival and proliferation of protozoan parasites during their intracellular mammalian stage. Since the ATPase activity executed in the N-terminal domain of HSP90 is critical for chaperone functions, HSP90 inhibitors capable to prevent ATP hydrolysis are expected to inhibit HSP90, leading to protein degradation and cell death, making this chaperone an attractive putative therapeutic target for cancer and leishmaniasis treatment (R Woodford *et al*., 2016; Palma *et al*., 2019; Batista *et al*., 2020). 17-*N*-Allylamino-17-demethoxygeldanamycin (17-AAG, tanespimycin) is an inhibitor of HSP90, which has been investigated in the treatment of cancer such as solid tumours and leukaemia. Alternatively, geldanamycin, and its analogues, 17-dimethylamino ethylamino-17-demethoxygeldanamycin (17-DMAG) and 17-AAG, may show a promising therapeutic activities against leishmaniasis (binding to the N-terminal domain of leishmanial-HSP90). However, the delivery of 17-AAG is difficult because of its poor aqueous solubility (Palma *et al*., 2019; Pires *et al*., 2020). Moreover, several molecules including Glb02, Glb08, Glb11, Glb16, Glb25 and Glb27 have been suggested as leishmanial-HSP90 inhibitors *via* binding to the N-terminal region of this protein (Batista *et al*., 2020).

### Aldehyde dehydrogenase (ALDH)

ALDHs are a group of enzymes found in all subcellular compartments that transform aldehydes to carboxylic acids. In *Leishmania*, ALDH is located in the mitochondrion (mALDH) and is overexpressed in the promastigote forms compared to the amastigotes (Saxena *et al*., 2007). It has been suggested as a protective protein against oxidative stress during glucose limitation in these parasites (Feng *et al*., 2011). Its expression significantly decreases in long culture of *Leishmania* and mALDH might be related to virulence (Magalhaes *et al*., 2014). Although different ALDH-isoenzymes have been identified in NSCLCs, the expression of such isoenzymes is unknown in *Leishmania* (Kang *et al*., 2016). The low expression of ALDH in normal IMR-90 human lung cells and in *Leishmania* spp. with attenuated infectivity may highlight the major role of ALDH in the pathogenicity of cancer and leishmaniasis (Bringaud *et al*., 1995; Chavali *et al*., 2008; Brocker *et al*., 2010; Feng *et al*., 2011; Kang *et al*., 2016). The high level of ALDH has been reported in drug-resistant cancer stem cells (Januchowski *et al*., 2013; Clark and Palle, 2016; Vassalli, 2019). It has also been shown that the use of *N*,*N*-diethylaminobenzaldehyde (DEAB) and a combination of gossypol (a pan-ALDH inhibitor) and phenformin leads to cancer cell death (Kang *et al*., 2016; Jiménez *et al*., 2019). The antiparasitic efficacy of gossypol, as an ALDH inhibitor, has been previously highlighted (Koppaka *et al*., 2012). A recent study has identified potential anticancer agents, as potent multi-ALDH isoform inhibitors, increased lipid peroxidation, ROS activity and toxic aldehyde accumulation, and also causing increased apoptosis and G2/M phase cell cycle arrest (Dinavahi *et al*., 2020). Such inferred data from cancer cells might clarify the possible function of ALDH in drug-resistant *Leishmania* strains and suggest the use of ALDH-isoform inhibitors not only for cancer treatment (Dinavahi *et al*., 2020), but also as a promising strategy for therapeutic assessments against leishmaniasis.

### Topoisomerases (TOPs)

TOPs are a group of enzymes that catalyse changes in the DNA topology during replication, transcription, recombination and genome repair. Firstly, they repeatedly can cut and join phosphodiester bonds from the phosphate deoxyribose structure which harbouring nitrogenous bases encoding genetic message. Secondly, they allow other DNA chains to pass between the temporary formed tails, by using energy from the nucleotide linkage and bind covalently to 3′ or 5′-DNA end (Wang, 2002; Pommier *et al*., 2016).

*TOP IB*: Type IB TOPs cut one DNA strand and covalently join to 3′ end. Two types of TOP IB inhibitors have been described: type I inhibitors or poisons, which stabilize the cleavage complex by creating a ternary complex DNA–enzyme drug; and type II inhibitors which act on the catalytic function of the enzyme. Despite their relevant role in genetic information fidelity conservation, TOP IB from *Leishmania* parasites are heterodimer enzymes which deeply differ from those of humans. For this reason, *Leishmania* TOP IB are considered as potential therapeutic targets. Camptothecins and their derivatives have demonstrated inhibitory activities against these parasitic enzymes (Prada *et al*., 2013). Synthetic indenoisoquinolines are potent TOP IB inhibitors with leishmanicidal activity ‘*in vivo*’ (Balaña-Fouce *et al*., 2012). Recently, hybrids of isoquinolines and camptothecins have shown their leishmanicidal activity through TOP IB activity inhibition (Reguera *et al*., 2019). Similarly, natural alkaloids and marine fatty acids have been reported as *Leishmania* TOP IB inhibitors (Carballeira *et al*., 2009, 2011, 2012*a*, 2012*b*, 2013; Chowdhury *et al*., 2011; Kumar *et al*., 2015, 2016; Pérez-Pertejo *et al*., 2019) as well as anticancer compounds (Carballeira *et al*., 2016, 2018).

On the other hand, human TOP I is a monomeric enzyme which has been demonstrated being overexpressed in several cancers (Lynch *et al*., 2001; Berney *et al*., 2002; Gouveris *et al*., 2007; Kümler *et al*., 2015). Most of the marketed TOP inhibitors applied for cancer treatment target TOP type II. However, there are camptothecin derivatives approved such as irinotecan, topotecan and belotecan (Hevener *et al*., 2018) which use TOP IB as a target.

*TOP II*: Type II TOPs are homodimeric enzymes responsible for catalysing topological changes in the DNA by transitory break of both nucleotide chains. During this process an intermediate covalent, between 5′-ends and such enzymatic subunits, is formed. Those proteins are conserved in blood parasites such as *Plasmodium*, *Trypanosoma* or *Leishmania* and their mammal hosts. However, the emerging interest on *Leishmania* TOP II was mainly due to its involvement in the kinetoplast DNA network and its replication. Moreover, TOP II is related to drug resistance in these parasites (Jayanarayan and Dey, 2003; Sengupta *et al*., 2005; Singh *et al*., 2009). The overexpression of a TOP II-like enzyme activity has highlighted the regulatory function of this putative enzyme in arsenite-resistant *L. donovani* strains (Jayanarayan and Dey, 2003). A point mutation, R250G, has been detected in the ATPase domain of the TOP II in arsenite-resistant strain of *L. donovani* parasite. The variation in the *TOP II* gene sequence between arsenite-sensitive and -resistant strains is anticipated to be responsible for the varied behaviour of this enzymes in response to antileishmanial/anti-TOP II agents (Singh *et al*., 2009). As aforementioned, similarly to TOP IB targeting agents, there are two groups of TOP II inhibitors depending of their mode of action, and some of them are even able to target both, type I and type II TOPs (Ray *et al*., 1997). For instance, isobenzofuranone derivatives are capable to inhibit *Leishmania* TOP II linked to DNA (Mishra *et al*., 2014; Chowdhury *et al*., 2018), whereas protoberberine perform its effect by stabilizing TOP II–DNA cleavage complex (Marquis *et al*., 2003). Mitonafide had demonstrated its activity on *Leishmania* nuclear and kinetoplast-TOP II (Slunt *et al*., 1996). Recently, a mitonafide derivative has shown a *Leishmania* TOP II inhibitory effect similar to type I inhibitors and at the mRNA level (Fernández-Rubio *et al*., 2019).

Human TOP II presents two isoforms: alpha (TOP 2A) and beta (TOP 2B) which exhibit differences in their molecular weight, genetic regulation and the location of the active site. TOP II expression in cancer lines has been largely studied (Doyle, 1994). Although TOP 2B isoform is expressed relatively constant throughout the cell cycle in both, normal and transformed cells, TOP 2A has been found abnormally expressed in different cancers such as breast, lung or prostate among others (Giaccone *et al*., 1995; Depowski *et al*., 2000; Mrklic *et al*., 2014; Schaefer-Klein *et al*., 2015; An *et al*., 2018; Liu *et al*., 2018). In fact, there are marketed anticancer drugs targeting TOP II, such as anthracycline and podophyllotoxin derivatives (Hevener *et al*., 2018). Etoposide, which belongs to the last group, is the best known TOP II poison, stabilizing DNA cleavage complex. Currently, TOP II molecules continue being a promising therapeutic target against cancer, and numerous research projects have focused on the synthesis of new inhibitors (Liberio *et al*., 2015; Karelia *et al*., 2017; Jiang *et al*., 2018; Yamashita *et al*., 2018; Li *et al*., 2018*b*).

### Proliferating cell nuclear antigen

PCNA is a processivity factor for DNA polymerase delta (Pol *δ*) and epsilon (Pol *ɛ*). It also interacts with other proteins involved in cell-cycle progression which are not parts of the DNA polymerase complex. PCNA has demonstrated its role in the replication and repair of DNA, chromatin assembly and RNA transcription. Its importance in *Leishmania* pathology is related to its significant role in drug response in clinical isolates (Tandon *et al*., 2014). In addition, this protein is a potential therapeutic target against leishmaniasis since it showed susceptibility to be inhibited at the mRNA level by selenocompounds (Fernández-Rubio *et al*., 2015; Fernández-Rubio *et al*., 2019). It is known that PCNA is overexpressed in tumour cells, to adapt the high capacity of such cells to exhibit an uncontrolled replication (Naryzhny and Lee, 2007). Interestingly, there are several small molecules, including cell-penetrating peptides, targeting PCNA with promising results against breast cancer and other tumours. For instance, several compounds inhibit the association of PCNA and chromatin, resulting in apoptosis and DNA damage in prostate and lung cancer (Dillehay *et al*., 2014; Lu and Dong, 2019). A cell penetrating peptide had been described as caspase-dependent apoptosis inductor which increased the activity of antitumour treatments in multiple myeloma cells (Muller *et al*., 2013).

### Tubulins

Tubulins are highly conserved dimeric proteins present in all eukaryotes. Alpha-beta (*α*/*β*) dimers polymerize to form microtubules, which serve as a skeletal system for living cells and participate in several essential functions, such as mitosis or intracellular transport among others (Montecinos-Franjola *et al*., 2019). In *Leishmania*, *α*-tubulin is a key component of the cytoskeleton, responsible for cell shape and involved in cell division, ciliary and flagellar motility (Ramírez *et al*., 2013). In addition, it has been related to drug resistance (Prasad *et al*., 2000; Jayanarayan and Dey, 2004). Furthermore, proteomic analyses demonstrated that this protein is more abundant in Sb(III)-resistant *Leishmania* cell lines (Matrangolo *et al*., 2013). Due to its role in *Leishmania* biology and pathology, *α*-tubulin has been considered a promising target against leishmaniasis and, consequently, compounds targeting this protein have been tested. Selenocompounds were able to significantly reduced *α-tubulin* gene expression (Fernández-Rubio *et al*., 2015). Podophyllum derived toxins are *Leishmania* tubulin inhibitors however, those showed discrepancies between protein activity and parasite growth inhibition (Escudero-Martínez *et al*., 2017). Such results differ from those of colchicine against trypanosomatids. This potent inhibitor of tubulin polymerization in higher eukaryotes seems to lack activity against these parasites, probably due to conformational changes in the protein which block colchicine access (Luis *et al*., 2013).

Alterations in the expression of tubulin are related to drug resistance in different cancers, including breast, lung, ovarian, gastric and prostate (Bernard-Marty *et al*., 2002; Hwang *et al*., 2013; Jiang *et al*., 2013; Tsourlakis *et al*., 2014; Du *et al*., 2015). Depending of their mechanism of action, tubulin inhibitors could be mainly grouped as microtubule-destabilizing agents or microtubule-stabilizing agents (Perez, 2009). The firsts are colchicine analogues and vinca alkaloids. The seconds are paclitaxel derivatives. Colchicine analogues bind to the colchicine binding site (CBS), one of the most important pockets for potential tubulin polymerization destabilizers. These compounds inhibit tubulin assembly and suppress microtubule formation (Lu *et al*., 2012). Nevertheless, currently there are not FDA (Food and Drug Administration) approved tubulin inhibitors targeting the CBS (Li et al., 2018*a*, 2018*b*). Taxanes, such as paclitaxel and its derivatives, bind to the interior surface of microtubules, resulting in their stabilization. Therefore, microtubules stabilization increase, leading to cell cycle arrest and apoptosis (Jordan, 2002; Jordan and Wilson, 2004).

### Voltage-dependent anion-selective channel protein 1 (VDAC-1)

VDAC-1 forms a large channel in the outer mitochondrial membrane that allows the diffusion of hydrophilic molecules. Apoptosis, metabolic flux and intracellular signalling are also important functions of this porin in eukaryotes. *Leishmania* parasites use anionic voltage-dependent channels as a transport system for adaption to nutritional stress conditions and pH homoeostasis (Vieira *et al*., 1994; Lawen *et al*., 2005; Shoshan-Barmatz *et al*., 2006; Bayrhuber *et al*., 2008).

In cancer cells, VDAC-1 is a protein with dual function involved in the regulation of survival and mitochondria-mediated apoptosis (Shoshan-Barmatz *et al*., 2017). It has been shown that the use of VDAC-1-specific small interfering RNA leads to the metabolism alteration and the growth suppression of cancer cells. Moreover, the up-regulation of the VDAC-1 increases the expression of apoptotic proteins such as hexokinase (HK), B-cell lymphoma-xL (Bcl-xL) and Bcl-2 in cancer cells and leads to the growth inhibition of such cells. Ion channel blockers have demonstrated activity against ion channel proteins in both, cancer cells and *Leishmania* parasites (Ponte-Sucre *et al*., 1998; Kale *et al*., 2015; Leanza *et al*., 2016; Reimão *et al*., 2016; Shoshan-Barmatz *et al*., 2017). Verapamil and nifedipine have been identified as human calcium channel blockers in cancer therapy which had been proposed to have mild anti-leishmanial activity (Kashif *et al*., 2017). *Leishmania donovani* Ca^2+^ ion channel (Ld-CC) has been suggested as potential drug target in leishmaniasis treatment (Kashif *et al*., 2017). Ld-CC regulates Ca^2+^ concentration which is involved in several functions such as mitochondrial oxidative metabolism and entry inside the macrophages and flagellar motion. Two ligands, ZINC17287336 and ZINC29590262 were showed highest binding affinity towards Ld-CC. These selected compounds have relatively more binding affinity than verapamil and nifedipine. Since ZINC29590262 has shown poor binding affinity towards the human voltage-dependent L-type calcium channel subunit alpha-1C in comparison with the Ld-CC, this compound can be suggested as an appropriate drug target (40% more binding affinity with Ld-CC than the human-voltage-dependent calcium channel) (Kashif *et al*., 2017).

### Mitochondrial import receptor subunit (TOM-40)

TOM-40 is located at the core of the translocase of the outer membrane (TOM) structure. Data produced by genome sequencing in protozoa has indicated the presence of TOM-40 homologues in *Cryptosporidium* (Keithly *et al*., 2005; Umejiego *et al*., 2008). Recent studies have reported TOM-40 in *L. infantum* amastigotes, and a protein with low similarity to TOM-40 in *Trypanosoma* (Zarsky *et al*., 2012; Rashidi *et al*., 2019). Although, the function of TOM-40 in *Leishmania* is unknown, bioinformatics data of the *T. brucei* genome for both TOM-40 and VDAC have identified a single open reading frame, with sequence analysis suggesting that TOM-40s and VDACs are ancestrally related and should be classified into the same protein family (the mitochondrial porins) (Pusnik *et al*., 2009). This information might open an attractive insight about using ion channel blockers against both TOM-40s and VDACs in Kinetoplastida such as *Trypanosoma* and *Leishmania* parasites.

As a tumour marker, TOM-40 is up-regulated in ovarian cancer cells and induces the proliferation and metastasis of these cells ‘*in vitro*’. It seems that TOM-40 increases the replication of cancer cells through regulating the mitochondrial activity and improving cellular energy and redox status (Yang *et al*., 2020). Evidence has shown that the inhibition of TOM-40 expression in ovarian cancer cells leads to a reduction in the proliferation and migration of cancer cells. However, directly targeting TOM-40 may be challenging in clinical application due to its substantial expression in normal cells. Since metformin (first-line therapy for type 2 diabetes) has been already clinically used with lower side-effects, this compound can be an appropriate alternative drug for targeting TOM-40 and the mitochondria (inhibiting mitochondria complex I) in epithelial ovarian cancer (Yang *et al*., 2020). The pathogenic functions of TOM-40 and therapeutic strategy against this target in cancer treatment might persuade the scientists to further investigate the expression and possible functions of TOM-40 in pathogenicity of leishmaniasis.

### Ornithine aminotransferase (OAT)

Aminotransferase are important enzymes that are able to transaminase aromatic amino acids. The functions of OAT are related to l-arginine pathways involved in polyamines production (Muxel *et al*., 2018). Polyamines metabolism is strongly important for *Leishmania* cell proliferation and infection (Ilari *et al*., 2015). For instance, a recent study has evaluated the effect of polyamine depletion in *L. donovani* mutants lacking ornithine decarboxylase or spermidine synthase. As mentioned in Table 1, DFMO inhibitor of ornithine decarboxylase, the enzyme that catalyses putrescine biosynthesis. Those results suggested that putrescine is not only a precursor metabolite for spermidine formation; it had specific functions for parasite viability and proliferation. These results also elucidated that ornithine decarboxylase inhibition and putrescine depletion was the most promising strategy for targeting polyamine biosynthetic pathway. It seemed that both polyamines (ornithine decarboxylase or spermidine) were required for parasite survival but that the presence of either putrescine or spermidine alone may allow *Leishmania* parasites to survive in a quiescent-like state for several weeks (Perdeh *et al*., 2020).

OAT as a *β-catenin* target gene in the liver is involved in the metabolism of glutamine. It has been shown that in hepatocellular carcinoma (HCC), the expression of OAT is up-regulated and the mechanism of this gene up-regulation is related to the activation of *β*-catenin signalling (Cadoret *et al*., 2002; Colnot *et al*., 2004). This information proposed that OAT, *β*-catenin signalling and the metabolism of glutamine are important factors in carcinogenesis especially in HCC (Cadoret *et al*., 2002; Thompson and Monga, 2007). The existence of inhibitors targeting OAT used to block the proliferation of HCC, may also allow the selection of this transferase as a therapeutic target against *Leishmania* parasites (Zigmond *et al*., 2015).

### Selenoproteins and selenoamino acid

Selenoproteins are a group of enzymes bearing selenocysteine in their catalytic domain. Many of the Se-bearing proteins participate in oxidative stress protection as observed in *Leishmania* parasites (Iribar *et al*., 2003; Da Silva *et al*., 2014). The use of leishmanial selenoproteins and selenoamino acid (selenomethionine) as therapeutic targets due to their role in modulation and evasion of the host immune responses has been recently suggested (Rashidi *et al*., 2020*a*, 2020*b*).

The role of selenoproteins and their metabolites including methylselenol, selenodiglutathione, Se-methylselenocysteine and selenomethionine has been underlined in the metabolism of lung cancer cells (Seng and Tiekink, 2012). Such metabolites inhibit protein kinases and alter cell cycle in cancer cells. Furthermore, these metabolites induce the activity of lymphokine-activated killer cells and natural killer cells and finally stimulate the immune system against cancer cells (Seng and Tiekink, 2012). The use of leishmanial selenoproteins inhibitors as well as the anti-tumour property of selenoproteins and their metabolites against cancer cells might suggest that such aforementioned compounds represent therapeutic targets for the treatment of leishmaniasis and cancers. Auranofin is gold-containing compound with well-known selenoproteins synthesis inhibitor properties. Its activity has been demonstrated against thioredoxin reductase (TrxRd), a Se-bearing enzyme involved in maintaining the intracellular redox state. In cancer, TrxRd overexpression is related to the aggressiveness of the malignancy (Kahlos *et al*., 2001; Lincoln *et al*., 2003) and its inhibition lead to apoptosis of tumour cells (You and Park, 2016). Using drug repurposing strategy, auranofin had been tested against parasitic diseases including *Leishmania*, with promising results. However, this compound seems not to target selenoproteins in these parasites, but rather interact with trypanothione reductase, a key enzyme of *Leishmania* polyamine-dependent redox metabolism (Ilari *et al*., 2012; Sharlow *et al*., 2014; Manhas *et al*., 2016). Nevertheless, the existence of specific selenoproteins inhibitors with effective activities against cancer cells might support the role of leishmanial selenoproteins as therapeutic targets (Yan *et al*., 2015; Arnér, 2017).

### Phosphoglycerate kinase-1 [PGK-1 (PKG-B)]

PGKs are transferases involved in ATP production from ADP and 1,3-diphosphoglycerate. Their involvement in the glycolytic pathway and survival of *Leishmania* parasites has been previously reported (Hart and Opperdoes, 1984; Blattner *et al*., 1992; Azevedo *et al*., 2015). PGKs (PGK-B) are overexpressed in antimony-resistant strains of *Leishmania*. Therefore, they might be related to the pathogenicity of these parasites (Blattner *et al*., 1998; Kazemi-Rad *et al*., 2013). Increased level of glycolysis enzymes such as PGK in the antimony resistant *Leishmania* isolates suggesting resistant strains require higher energy to protect against antimony-induced oxidative stress. Alternatively, the overexpression of such enzyme might lead to enhancing in pyruvate which can remove peroxides and participate to reduce oxidative stress (Biyani *et al*., 2011). In both, *Leishmania* and mammalian cells, PGKs are encoded by two genes: *gene B* and *gene C*, and *PGK-1* and *PGK-2*, respectively (Watson and Littlechild, 1990; McKoy *et al*., 1997). Several monosubstituted N6 and N2 adenosine derivatives were selected to screen against *T. brucei* PGK. Of these, 2-amino-N6-substituted analogues represented appropriate activity against the parasite kinase compared with the N6 compounds that lacked the C2 amino group, although activity was still weak (Merritt *et al*., 2014). Since protein kinase inhibition has been primarily discussed as anti-trypanosomatid strategy in treatment, these proteins such as PGK can further investigated in leishmaniasis.

The importance of PGK-1 in cancer development resides on its involvement in drug-resistance and its dual action depending on the cellular environment. Under intracellular hypoxia conditions, it plays an oncogenic role. However, it decreases tumour growth when it is secreted extracellularly through angiogenesis inhibition (Daly *et al*., 2004; He *et al*., 2019). In addition to the metabolic functions of PGK-1 in cancer cells, this enzyme induces and increases the angiostatin formation and leads to the restriction of angiogenesis in tumours. The anti-tumour property of PGK-1 has been shown in Lewis lung carcinoma (LLC-1). It is well known that cyclooxygenase-2 (COX-2), as an important marker of resistance to apoptosis in cancer cells, promotes angiogenesis and metastasis (Tsujii and DuBois, 1995; Tsujii *et al*., 1998). Due to the overexpression of PGK-1 in LLC-1, COX-2 is decreased and therefore, cell invasion, prostaglandin E2 and angiogenesis are affected by this mechanism. Finally, the progression of cancer cells is then restricted (Tang *et al*., 2008; Ho *et al*., 2010). Under solid tumours and hypoxia conditions, PGK-1 is the main source of production of ATP. Solid tumour cells use mechanisms that inhibit the production of PGK and decrease the angiostatin formation. Furthermore, other angiogenesis activators such as vascular endothelial cell growth factor are active in solid tumours (Daly *et al*., 2004). Currently, potential PGKs-inhibitors are under development (He *et al*., 2019).

## Conclusion

The up- and down-regulation of the aforementioned proteins were mostly related to the survival, development, pathogenicity, metabolic pathways and vital signalling in *Leishmania* parasites and cancer cells. As an interesting issue, the regulation of these markers can be investigated in interactions that can be occurred between *Leishmania* parasites and cancer cells under ‘*in vivo*’ and ‘*in vitro*’ conditions. The reliable validation of the expression of such proteins in *Leishmania* parasites and cancer cells using further experiments is warranted to subsequently confirm their possible functions. Further investigation of the differentially regulation of common expressed proteins between cancer cells, normal human cells, low-pathogenic and high-pathogenic forms of *Leishmania* parasites might elucidate novel and attractive information concerning such proteins. The existence of common triggering factors reflects mutual features in the etiopathogenetic mechanisms underlying leishmaniasis and cancer. Given these similarities, lessons learned from strategies against cancer may be relevant to design adequate approaches to reduce and eliminate leishmaniasis. Herein, we focused specifically on the shared mechanisms at protein scale. Taken together, the introduction of common expressed proteins in *Leishmania* parasites and cancer cells might reveal valuable information regarding the possible common mechanisms of pathogenicity and therapeutic targets in leishmaniasis and cancers. Taking into account that current therapies for neglected diseases are based in drugs lacking effectiveness, the lack of new specific anti-*Leishmania* compounds and of research focused on this group of diseases, drug repurposing constitutes a useful tool to find effective candidates in leishmaniasis control and elimination. This review reinforces the likely functional similarities between many proteins in cancer and parasites, some of them being recognized therapeutic targets and thus the potential use of drugs with proven efficacy in the treatment of cancer for treating parasitic diseases and vice versa, opening new avenues to the one health approach.
